# MAXIMIZING COLLECTIVE IMPACT ACROSS THE AGE-FRIENDLY ECOSYSTEM

**DOI:** 10.1093/geroni/igad104.0331

**Published:** 2023-12-21

**Authors:** Holly Dabelko-Schoeny, Teri Kennedy

## Abstract

Age-friendly initiatives have the potential to transform communities through inclusive policies and practices that meet the needs of people, families, and communities across ages and abilities. The ecosystem of age-friendly initiatives (communities, universities, public health systems, and health systems) has grown exponentially over the last decade. Despite shared values and principles, each age-friendly initiative largely operates independently with limited opportunities to share knowledge to maximize collective impact and social change. This theoretically-driven symposium provides an overview of each age-friendly initiative, challenges faced, and ideas for innovation within and across each initiative. Dabelko-Schoeny and Sheldon will utilize a macro application of the KAP (knowledge, attitude, practice) theory of behavior change to identify the vast opportunity for synergies and grand challenges across the age-friendly ecosystem. Greenfield will provide an overview of the development of the Age-Friendly Cities and Community Movement, and present ideas regarding challenges and opportunities for the future based on insights from an ongoing, community-engaged study in New Jersey. Montepare will describe the Age-Friendly University initiative and how age-inclusive practices in higher education can extend and enhance ecosystem efforts. Phillips and Wolfe will provide examples of Age-Friendly Public Health Systems including a description of a collective impact approach for state-wide age-friendly policy and practice adoption. Finally, Kennedy will offer approaches and models to advance the adoption and sustainability of age-friendly principles, programs, and policies by leveraging synergies and implementation science. This is an Age-Inclusivity in Higher Education Interest Group Sponsored Symposium.

